# Computational Approach for Spatially Fractionated Radiation Therapy (SFRT) and Immunological Response in Precision Radiation Therapy

**DOI:** 10.3390/jpm14040436

**Published:** 2024-04-21

**Authors:** Paolo Castorina, Filippo Castiglione, Gianluca Ferini, Stefano Forte, Emanuele Martorana

**Affiliations:** 1 Istituto Oncologico del Mediterraneo, Via Penninazzo, 7, 95029 Viagrande, Italy; stefano.forte@grupposamed.com (S.F.); emanuele.martorana@grupposamed.com (E.M.); 2 INFN, Sezione di Catania, Via Santa Sofia, 64, 95123 Catania, Italy; 3 Faculty of Mathematics and Physics, Charles University, V Holešovičkách 2, 18000 Prague, Czech Republic; 4 Biotechnology Research Center, Technology Innovation Institute, Abu Dhabi P.O. Box 9639, United Arab Emirates; filippo.castiglione@tii.ae; 5 Institute for Applied Computing, National Research Council of Italy, Via dei Taurini, 19, 00185 Rome, Italy; 6 REM Radioterapia, Via Penninazzo, 11, 95029 Viagrande, Italy; gianluca.ferini@grupposamed.com

**Keywords:** in-silico model, radiotherapy, immunotherapy, Spatially Fractionated Radiation Therapy, mathematical framework

## Abstract

The field of precision radiation therapy has seen remarkable advancements in both experimental and computational methods. Recent literature has introduced various approaches such as Spatially Fractionated Radiation Therapy (SFRT). This unconventional treatment, demanding high-precision radiotherapy, has shown promising clinical outcomes. A comprehensive computational scheme for SFRT, extrapolated from a case report, is proposed. This framework exhibits exceptional flexibility, accommodating diverse initial conditions (shape, inhomogeneity, etc.) and enabling specific choices for sub-volume selection with administrated higher radiation doses. The approach integrates the standard linear quadratic model and, significantly, considers the activation of the immune system due to radiotherapy. This activation enhances the immune response in comparison to the untreated case. We delve into the distinct roles of the native immune system, immune activation by radiation, and post-radiotherapy immunotherapy, discussing their implications for either complete recovery or disease regrowth.

## 1. Introduction

The Spatially Fractionated Radiation Therapy (SFRT) techniques, based on a highly heterogeneous radiation precision delivery, spatially alternating low (valleys) and high (peak) doses within the tumor, are currently under intense investigation [1]. Two main forms, the GRID and the Lattice ones, have been applied clinically [2]. They differ for the inhomogeneous dose distribution pattern, which is geometrically arranged in the GRID and more randomly oriented in the Lattice technique [3]. The latter, depending on the possibility of detecting the oxygen background of the tumor or its surrogates, is more suitable to be planned to selectively target the most radioresistant hypoxic tumor subvolumes with high peak doses [4,5]. In this respect, two unconventional approaches of irradiation have been recently proposed for partially treating bulky tumors exhibiting an inhomogeneous energetic metabolism as reflecting varied oxygenation across different tumor areas: the Stereotactic Body Radiotherapy targeting Partial Tumor Hypoxic clonogenic cells (SBRT-PATHY), codified by Tubin et al. [6], implies a homogeneous high-dose irradiation of the whole hypoxic tumor subvolume while sparing the normoxic one, whereas the metabolism-guided Lattice technique developed by Ferini et al. involves an even more limited irradiation of the hypoxic volume by segmenting within it small spheres of high doses (vertices) acting as potentially trigger points to elicit bystander effects on the unirradiated adjacent tumor tissues [7]; although, these phenomena have been also described in non-tumor healthy tissues [8]. The effectiveness of both techniques likely relies on the host immune system recruitment, which would be reprogrammed to fight against cancer cells [9,10]. Moreover, the model by Ferini et al. could be more advantageous from the point of view of the tolerability profile, as it is probably characterized by a less toxic impact on the healthy tissues surrounding the tumor thanks to a more favorable dose-volume effect compared to SBRT-PATHY [7]. It is well known that radiotherapy induces anti-tumor immunity and an extreme example of immune activation by radiotherapy is the phenomenon known as the “abscopal effect”, i.e., the regression of a metastatic tumor distantly located from the irradiated tumor [11]. In this letter, we proposed a computational approach of the combined effects of Lattice radiotherapy, with an initial localized high dose, and of the triggered immune response which originates from the clinical findings of a case report [7]. However, the mathematical formulation is completely general. Indeed, one quantitatively analyzes the tumor evolution, after the first higher dose in some specific sub-areas of its mass, including the regrowth pattern and the effect of the activation, which is also taken into account during the standard low-dose palliative radiotherapy protocol. Finally, the effect of immunotherapy, starting at the end of radiotherapy, is included. The critical conditions for a stable disease, complete recovery or recurrence are discussed.

These quantitative evaluations of the immune response induced by radiation and the passive immunotherapy, represent an important step forward compared to [7], where clinical aspects are discussed, and the role of the immune system remains qualitative.

## 2. The Case Report: Radiotherapy and Clinical Results

**Figure 1:**
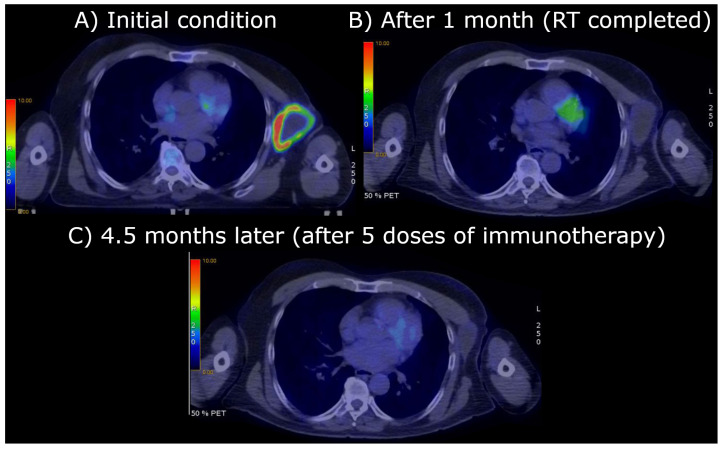
^18^F-FDG PET at different time intervals showing the biggest metastasis located at left axilla: (**A**) starting condition (171.3 cm^3^); (**B**) after 1 month and the end of radiotherapy; (**C**) after 4.5 months and 15 weeks of immunotherapy with cemiplimab (5 intravenous injections).

**Figure 2:**
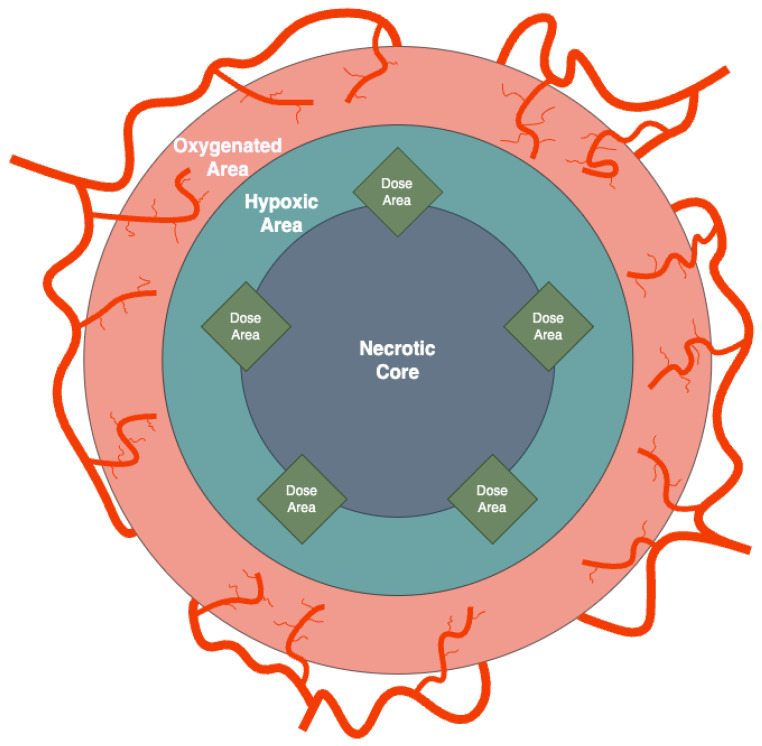
Graphical representation of the initial tumor setting. The gross tumor volume (GTV) is divided in three concentric areas: death cells (necrotic), non-proliferating tumor cells (hypoxic) and the cancer cells proliferating zone (oxygenated) with blood vessels.

**Figure 3:**
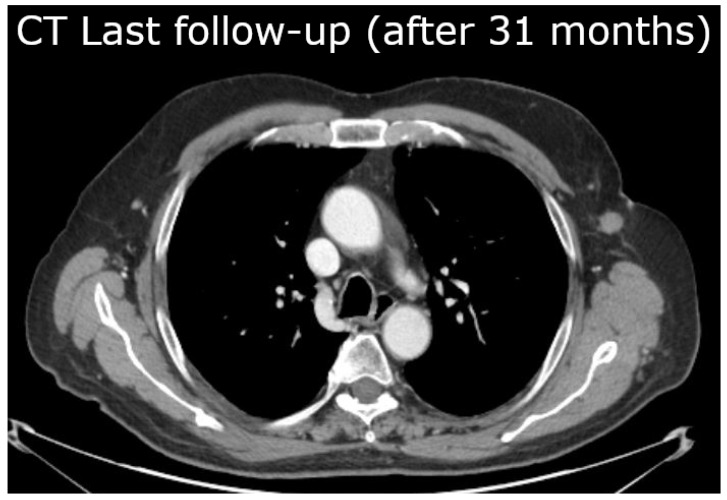
CT performed in the last follow-up (November 2023) focused on the axillary zone, the site of a metastasis with size 171.3 cm^3^ (April 2021), shows no cancer recurrence. The patient was still under immunotherapy.

A 75-year-old patient with a history of facial squamous cell carcinoma resected in December 2017 developed three metastases in February 2021: one located at the II left sternocostal joint (3.5 cm maximum diameter) and two lymphadenopathies located at the neck and left axilla (2 cm and 10.4 cm, respectively) [1,2,3]. The two smallest metastases were treated with stereotactic body radiotherapy (SBRT) at a dose of 30 Gy in five fractions of 6 Gy/day delivered homogeneously to all tumor components, whereas the bulky axillary one, Figure 1A, (171.3 cm^3^) was irradiated inhomogeneously with a spatially fractionated radiotherapy (SFRT) technique based on an ^18^F-FDG PET-guided segmentation reflecting different oxygenation patterns within the tumor tissue as Figure 1A highlights [4,5]: the photopenic core and the ^18^F-FDG-avid external rim on the sides of a suspected hypoxic mid-layer (13 cm^3^) were postulated as necrotic (86.8 cm^3^) and well oxygenated (71.5 cm^3^), respectively. To better understand the stratification we have reported a generalization of the segments in Figure 2. The SFRT protocol involved a first phase, in which a single shot of 15 Gy was delivered to five 1 cm diameter vertices delineated in the hypoxic volume, followed by a palliative dose (30 Gy in 10 fractions of 3 Gy/day) homogeneously targeting the entire metastasis. One month later, immediately before starting passive immunotherapy with cemiplimab, an early reassessment of the overall tumor burden by ^18^F-FDG PET documented a complete metabolic response of the axillary lesion, shown in Figure 1B, and a partial response and stable disease of the other two metastases (the ones treated with SBRT), respectively. In September 2021, after 20 weeks from the end of radiotherapy and upon the completion of 15 weeks of immunotherapy with cemiplimab, Figure 1C, a further ^18^F-FDG PET was negative. At the last follow-up in November 2023, the patient was in good health and still in the course of treatment with immunotherapy; the assessment of the CT scan shown in Figure 3 confirms the absence of tumor relapse.

## 3. Methods

The treatment of the massive axillary tumor can be discussed quantitatively, and the general computational model, resulting from the clinical case discussed, is given in Appendix A. The image segmentation technique applied to the pre-treated tumor volume is represented in Figure 2 where different cancer zones involved in the model can be identified.

The tumor volume has a spheroid-like geometrical setting where the central zone represents necrosis, under a layer of cells with a deficit of oxygenation (hypoxia) and surrounded by a normal oxygenated area (normoxia); the SFRT targeting the hypoxic segment in five vertices that overlap partially with the necrotic one.

The linear quadratic model has been applied to determine the radiotherapy effect, including the oxygen enhancement ratio (OER) for hypoxic cells, and the effects of the initial large dose on the well-oxygenated areas propagate, with different effects, up to the corona external border. Untreated tumor (re)growth according to the Gompertz law (GL) (other growth laws can also be used) and the immune radiobiological effects have been included in the tumor progression during therapy. At the end of radiotherapy, the time evolution follows the Gompertz modified by immunotherapy.

These computational methods result in a procedure useful to drive clinical decisions and gain insight into the evolution of the disease (mathematical details in Appendix A). More precisely, let us define some crucial notions to better understand the mathematical model:

- $V(1^{-})$ was defined as the tumor volume before the first treatment;
- $V_{exp}\left(n^{+}\right)$ is the observed volume after *n* doses (“exp” indicates the experimental value);
- $V_{th}^{rad}\left(n^{+}\right)$ is the volume numerically evaluated starting with $V(1^{-})$ by applying the Linear Quadratic Model (LQM) (including OER when necessary) for *n* doses (“rad” indicates radiotherapy).

**Figure 4:**
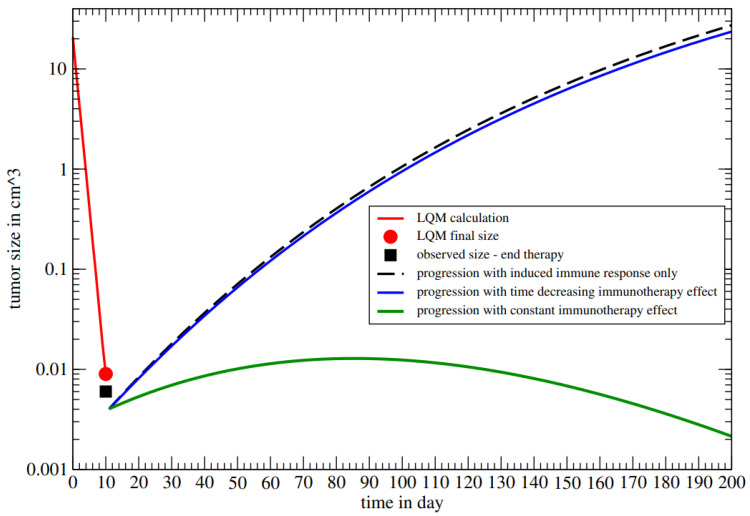
Normoxic volume progression. Red curve: LQM calculation with endpoint at 0.009 cm^3^. Black square: experimental tumor size at the end of radiotherapy (assumed 0.006 cm^3^). Black curve: tumor regrowth with induced immune response, A(0,10)=0.4 and no immunotherapy. Blue curve: tumor regrowth with A(0,10) plus a slow increasing immunotherapy *B*(*t*,11) = 0.05 *ln* (*t*/11). Green curve: complete recovery due to A(0,10) plus a constant I(t) which gives a linear increase with time of the cumulated effect *B*(*t*,11) = 0.05 (*t*− 11). *t* in day.

Therefore some useful clinical evaluations are obtained from the following experimental and quantitative processes:

1. Measurement of the initial tumor volume $V(1^{-})$, since the initial size of the untreated tumor includes the effect of the host immune response.
2. Numerical evaluation of the final volume according to the scheduled radio-treatment and the LQM (including OER if necessary). For example, for the normoxic cell volume, one gets
  (1) $$V_{th}^{rad}\left(n^{+}\right)=V\left(1^{-}\right)exp\left[\left(-\alpha d-\beta d^{2}\right)n\right]$$
  after *n* treatments of dose *d*, with time interval Δt=1 day, and assuming the regrowth is negligible between daily subsequent doses (the regrowth is however included in the mathematical formulation, see Equations (A5) and (A6) of Appendix A. $V_{th}^{rad}$ is the theoretical, expected, tumor shrinkage by radiotherapy only.
3. Measurement of the final tumor volume $V_{exp}\left(n^{+}\right)$, after *n* doses, which gives the effective tumor volume reduction.
4. Comparison of the effective tumor size at the end of therapy with the theoretical value. If $V_{exp}\left(n^{+}\right)<V_{th}^{rad}\left(n^{+}\right)$, define
  (2) $$\Delta V=\frac{V_{exp}\left(n^{+}\right)}{V_{th}^{rad}\left(n^{+}\right)},$$
  which describes the difference between the observed volume and the expected one by LQM, at the end of radiotherapy.
5. According to the computational model, ΔV<1 is due to the immune response activated by radiotherapy, *A* (see Equations (A5) and (A6) in Appendix A), which at the end of the *n* treatments turns out to be
  (3) A(0,nΔt)=−lnΔV.
  *A* (0, *n*Δ*t*) quantitatively defines the induced immune response. In other words, the activation of the immune response due to radiotherapy (to note, this is not a “passive or exogenous” immunotherapy, but a consequence of the immune response to the cell debris resulting from apoptotic cells due to radiation) determines, at the clinical level, the difference between the “theoretical value” calculated by the LQM and the observed tumor size reduction.
6. To estimate the specific regrowth rate at the end of radiotherapy (see Equations (A8) and (A9) in Appendix A) one needs to evaluate the constant
  (4) $$ln(\frac{V_{\infty }}{V_{exp}\left(n^{+}\right)})$$
  where $V_{\infty }$ is the lethal maximum tumor volume supported by the environmental condition (oxygen, nutrient supplies, …), generally corresponding to $10^{12}$ cells, i.e., about a diameter of 12 cm [12].
7. Compare the previous constant with the calculated A(0,nΔt) in Equation (3). If
  (5) $$ln\left(\frac{V_{\infty }}{V_{exp}\left(n^{+}\right)}\right)-A\left(0,n\Delta t\right)<0,$$
  then the disease evolves towards complete recovery, due to the immune response activated by radiotherapy, because the specific rate turns out to be negative. In general, this is not the case, since the first term is large.
8. More precisely, immediately after the end of radiotherapy, according to the reasonable assumption that in this limited timeframe the induced immune response remains almost constant, the progression depends on the condition
  (6) $$ln\left(\frac{V_{\infty }}{V_{exp}\left(n^{+}\right)}\right)\left[1-exp\left(-k\left(t-n\Delta t\right)\right)\right]-A\left(0,n\Delta t\right)<0.$$
  A few days, *m*, after the end of radiotherapy (in such a way kmΔt<<1), the previous condition can be written as
  (7) $$km\Delta tln\left(\frac{V_{\infty }}{V_{exp}\left(n^{+}\right)}\right)-A\left(0,n\Delta t\right)<0$$
  that can be calculated since the GL parameter *k* is experimentally known [13], and A(0,nΔt), previously evaluated, can be considered a reliable estimate of the activated immune response, due to the short time interval.
9. If the previous condition is verified, the tumor volume initially decreases after the end of therapy, but the progression can restart. Indeed, for t>nΔt, the time evolution without further immunotherapy follows the law in Equation (A7) of Appendix A (see also Figure 4).
  By assuming a constant effect of the radiotherapy-activated immune response following radiotherapy, the time for the beginning of the regrowth can be evaluated and it turns out to be (see Equation (A9) in Appendix A, for t=mΔt)
  (8) $${\left(m\Delta t\right)}_{regrowth}=-\frac{1}{k}ln\left[1-\frac{A(0,n\Delta t)}{ln(\frac{V_{\infty }}{V(n^{+})})}\right].$$
  On the other hand, for a more realistic, time-dependent immune response, the critical time is defined by the implicit relation
  (9) $$ln\left(\frac{V_{\infty }}{V(n^{+})}\right)\left[1-exp\left(-k\left(t_{regrowth}-n\Delta t\right)\right)\right]-A\left(t_{regrowth},n\Delta t\right)>0.$$
  In general, since the stronger constraint in Equation (5) is not satisfied, the patient needs immunotherapy to increase the possibility of a complete recovery or a late tumor regrowth. The effects of the immunotherapy, for t>nΔt, are described by the function B(t,nΔt), reported in Equations (A12) and (A13) in Appendix A. The condition of the previous Equation (6) for complete recovery now becomes
  (10) $$ln\left(\frac{V_{\infty }}{V(n^{+})}\right)\left[1-exp\left(-k\left(t-n\Delta t\right)\right)\right]-A\left(t,n\Delta t\right)-B\left(t,n\Delta t\right)<0.$$

In the next section, the previous computational model will be applied to the clinical case presented, with its specific setting.

## 4. Application to the Case Report

Let us now apply the previous procedure to the case report, where one has to take into account the geometrical aspects, related to the necrotic core, the normoxic, hypoxic subpopulations and the insert of vertices (see Figure 2).

The total tumor volume and necrotic core, hypoxic area and vertex volumes are initially measured (see Appendix A). Then, a first dose of 15 Gy has been administrated in the vertices, followed by the standard protocol of 10 daily doses of 3 Gy to the whole tumor. By the analysis of the specific setting, the calculations of the reduction in the active volumes (i.e., of the normoxic and hypoxic volumes), based on the LQM including OER, show that at the end of the complete radiation treatment, the normoxic volume has been reduced by the factor $1.19\times 10^{-4}$ (see Table A1 in Appendix A) and the hypoxic volume (OER =1.5) by $3.2\times 10^{-3}$. Since the initial normoxic volume is 71.5 cm^3^, its final volume, theoretically evaluated, turns out to be $71.5\times 1.19\times 10^{-4}≃0.009$ cm^3^. In the considered case report, the final normoxic volume (and the hypoxic one) is indeed very small and not detectable. Therefore, the comparison between the theoretical value and the observed one, which is the crucial point 4 of the procedure, cannot be exactly carried out.

However, for illustrative purposes, let us assume that the observed final volume of the normoxic area is ≃0.006 cm^3^, corresponding to a metabolic active volume of a diameter ≃2.5 mm, lower than the resolution of PET detection. Therefore, the cell killing due to the immune response activated by radiotherapy is given by (point 5)

(11) $$A\left(0,n\Delta t\right)=-ln\Delta V=-ln\left[\frac{V_{exp}\left(n^{+}\right)}{V_{th}\left(n^{+}\right)}\right]=-ln\left(6/9\right)≃0.4,$$

which is much smaller than $ln[V_{\infty }/V_{exp}\left(n^{+}\right)]$, indicating that the activated immune response is not enough to drive toward complete recovery (see Equations (5) and (7)). Indeed, immunotherapy after radiotherapy is crucial for the evolution of the disease, and one has to follow the tumor size progression according to the total immune response induced by radiotherapy plus immunotherapy, respectively, given by A(t,nΔt) and B(t,nΔt) in Equation (9).

The progression of the normoxic volume during and after radiotherapy is depicted in Figure 4 for different total immune responses.

At time (day) t=0, the normoxic volume has been reduced by the large localized dose of 15 Gy in the vertices. The red line represents the result of the LQM, by 30 Gy in 10 daily doses, reporting the reduced volume (Table A1 in Appendix A). As discussed, assuming that the experimental measure of the tumor size at the end of therapy gives 0.006 cm^3^, less than 0.009 cm^3^, *A* (0, 10 days) = 0.4. By considering the lethal tumor size of 1 liter [12], one gets

(12) $$ln\left(\frac{V_{\infty }}{V(n^{+})}\right)=ln\left(1000/0.006\right)≃12>>A\left(0,10\;\mathrm{days}\right).$$

Therefore, in this example, the most important role in the progression control originates from immunotherapy. Its cumulative effect, B(t,10) in Equation (A14), on the tumor size evolution, is reported in Figure 4 for a logarithmic slow increase, corresponding to the O(1/t) behavior of the function I(t) in Equation (A14) (blue curve), and for a linear time dependence (green curve), related to a constant immunotherapy effect per dose (I(t)= constant).

The last clinical follow-up (November 2023) shows the patient in good condition, without recurrences, which implies that the condition in Equation (10) has been satisfied, due to immunotherapy. Although the understanding of this result would require a microscopic model, the previous example suggests that the administrated immunotherapy (i.e., passive immunity) induces at least a constant effect (I(t)= constant) in tumor control.

For advanced cutaneous head and neck squamous cell carcinoma, chemo- and immunotherapy after radiotherapy are part of the general protocol. However, the suggested computational approach gives useful information, and immunotherapy is crucial for a stable disease or a complete recovery independently of the possible detection of the final active volumes.

## 5. Discussion and Conclusions

Some preclinical and clinical evidence confirms the synergistic action of radiotherapy (RT) and immunotherapy against the tumor cells [14]. Although the intrinsic sensitivity to radiation is patient-specific [15,16] and may depend on different factors, RT is able to ablate cancer cells not only by directly induced necrosis or apoptosis but also by triggering an immune response that actively recruits immune cells within the tumor microenvironment. For example, RT promotes the release of tumor-associated antigens, which, once processed by antigen-presenting cells (APCs), prime CD8^+^4 T cells in the draining lymph nodes.

The synergy between precision radiotherapy and the activation of the immune system for cancer treatment is an important aspect of clinical decisions at the end of therapy. Without a quantitative description of their combined effects, it is difficult to understand the final results of radiotherapy based on the LQM alone: the role of the activated immune system cannot be neglected.

The proposed quantitative method considered the immune activation due to non-homogeneous radiotherapy, according to the recent proposals involving limited irradiation of the hypoxic volume by segmenting it into small spheres of a high dose. This requires high-precision radiotherapy.

The mathematical machinery is rather simple, and steps 1–9 of Section 3 clarify the assumptions, which can be modified by considering specific settings (different growth laws, geometry, modulations …).

In particular, the computational algorithm gives a clear indication of the following aspects:

1. The evaluation of the cell killing fraction or volume shrinkage due to the immune response activated by radiotherapy, as a difference to the standard LQM results;
2. An estimate of the complete recovery condition or of the regrowth time, by considering a constant immune-activated response at the end of radiotherapy;
3. A possible prediction of the immunotherapy effects after the final radiation dose, by patient-oriented monitoring observations, which permits a phenomenological determination of the function *B* in Equations (10) and (A14).

We are aware of the importance of conducting rigorous experimental investigations to solidify the theoretical basis of our approach. By performing targeted studies and gathering more empirical data, one validates the significance of the proposed computational approach as a complementary, useful, tool to evaluate the possible tumor progression after radiotherapy and immune response.

The induced immunity response often attacks both primary tumor and metastatic sites, posing the biological basis of the in situ vaccination driving the so-called abscopal effect [17,18,19]: RT induces a systemic behavior that can activate the immune response against metastasis, i.e., in locations that are far from the RT-treated primary tumor. The abscopal effect is not included in the analysis, since it requires a devoted study.

Finally, the method is based, as a large part of the mathematical tools for tumor growth and therapy, on deterministic differential equations. On the other hand, the variability in microscopic biological conditions would require an analysis by stochastic differential equations to evaluate the probability of the different outcomes of the complete therapy. This aspect implies the generalization of the mathematical results in Appendix A.

## Data Availability

The data that support the findings of this study are available in ref [7].

## Appendix A

### Appendix A.1. General Formalism

The tumor progression during radiotherapy is a combination of the cell killing due to the radiation, described by the LQM, and of the regrowth in the time interval between two subsequent doses. However, the regrowth has been slowed by the immune response activated by radiotherapy. Therefore, between dose *i* and i+1, the evolution follows the modified GL which, for the homogeneous system, is the solution of the equation

(A1) $$\frac{1}{V}\frac{dV}{dt}=kln\left(\frac{V_{\infty }}{V}\right)-\gamma M\left(t\right)$$

where $V_{\infty }$ is the maximum tumor volume supported by the environmental condition (oxygen, nutrient supplies, …), M(t) describes the activated immune response, and γ is a constant. The general solution of the previous equation is

(A2) $$V\left(t\right)=V\left(t_{0}\right)e^{ln\left(\frac{V_{\infty }}{V_{0}}\right)\left[1-exp\left(-k\left(t-t_{0}\right)\right)\right]-A\left(t_{0},t\right)}$$

where $V_{0}$ is the initial ($t_{0}$) value and

(A3) $$A\left(t_{0},t\right)=\gamma \int_{t_{0}}^{t}{dt}^{\prime }M\left(t^{\prime }\right)e^{-k(t-t^{\prime })}.$$

According to the LQM, the single dose, *d*, reduces the volume by a factor of

(A4) $$S=e^{-[\alpha d+\beta d^{2}]}$$

where α and β are constants, experimentally determined and depending on the tumor phenotype.

Defining $V(1^{-})$, the observed volume before the first treatment, the final volume after *n* treatment, $V(n^{+})$, can be easily derived by the solution of the previous equation and an iterative procedure. One obtains

(A5) $$V\left(n^{+}\right)=V\left(1^{-}\right)e^{ln\left(\frac{V_{\infty }}{V1^{-}}\right)\left[1-exp\left(-kn\Delta t\right)\right]-A\left(0,n\Delta t\right)-LQ\left(n\right)}$$

where Δt is the time interval between two doses and

(A6) $$LQ\left(n\right)=\left(\alpha d+\beta d^{2}\right)\left[1+e^{\left(-k\Delta t\right)}+\ldots +e^{-(n-1)k\Delta t}\right]$$

is the effect of radiotherapy. Notice that without regrowth, i.e., k=0, this effect reduces to the usual LQM results.

The tumor evolution after the end of therapy, that is for time t>nΔt, is again the solution of the previous Gompertz equation with the initial condition V(n+):

(A7) $$V\left(t\right)=V\left(n^{+}\right)e^{ln\left(\frac{V_{\infty }}{V(n^{+})}\right)\left[1-e^{\left(-k(t-n\Delta t\right)}\right]-A\left(t,n\Delta t\right)}\;t>n\Delta t.$$

By assuming that the activated immune response does not change after the end of radiotherapy, for any t>nΔt, one obtains

(A8) A(t,nΔt)=A(0,nΔt)

which implies that the condition for a stable disease, a complete recovery or regrowth can be derived by the sign of

(A9) $$ln\left(\frac{V_{\infty }}{V(n^{+})}\right)\left[1-exp\left(-k\left(t-n\Delta t\right)\right)\right]-A\left(0,n\Delta t\right).$$

For example, if

(A10) $$ln\left(\frac{V_{\infty }}{V(n^{+})}\right)-A\left(0,n\Delta t\right)<0,$$

then one gets the complete recovery, which can be also obtained if at some time $t_{c}$, the condition

(A11) $$ln\left(\frac{V_{\infty }}{V(n^{+})}\right)\left[1-exp\left(-k\left(t_{c}-n\Delta t\right)\right)\right]-A\left(0,n\Delta \right)<0$$

is satisfied. On the other hand, if the previous exponent becomes positive (at some time), the tumor regrows.

### Appendix A.2. Including Immunotherapy

According to the previous discussion, Equations (A8)–(A10) give a criterion to verify the tumor progression after the end of radiotherapy. If the condition in Equation (A10) is not satisfied, the regrowth effect can overcome the immune response triggered by radiotherapy. In such a case, immunotherapy is extremely important to reach a stable disease or a complete recovery. The immunotherapy effect, I(t), modifies the Gompertz equation for t>nΔt, i.e.,

(A12) $$\frac{1}{V}\frac{dV}{dt}=kln\left(\frac{V_{\infty }}{V}\right)-\gamma M\left(t\right)-\delta I\left(t\right)$$

where δ is a constant, and the volume progression becomes (for t>nΔt)

(A13) $$V\left(t\right)=V\left(n^{+}\right)e^{ln\left(\frac{V_{\infty }}{V(n^{+})}\right)\left[1-e^{\left(-k(t-n\Delta t\right)}\right]-A\left(t,n\Delta t\right)-B\left(t,n\Delta t\right)}$$

where B(t,nΔt) is given by

(A14) $$B\left(t,t_{i}\right)=\delta \int_{t_{i}}^{t}{dt}^{\prime }I\left(t^{\prime }\right)e^{-k(t-t^{\prime })},$$

where $t_{i}$ is the initial time of the immunotherapy. The critical condition is now

(A15) $$ln\left(\frac{V_{\infty }}{V(n^{+})}\right)\left[1-exp\left(-k\left(t-n\Delta t\right)\right)\right]-A\left(0,n\Delta \right)-B\left(t,t_{i}\right)<0$$

which strongly requires starting the immunotherapy immediately after the end of radiotherapy.

### Appendix A.3. Geometrical Setting and Radiotherapy Treatment of the Case Report

According to the tumor spheroid model depicted in Figure 2, let us call $V_{T}^{i},V_{T}^{f},V_{c}^{i},V_{c}^{f},$$V_{n}^{i},V_{n}^{f}$$,V_{h}^{i},V_{h}^{f}$, respectively, the initial and final volumes of the total system, of the normoxic corona, of the necrotic core and of the hypoxic corona.

The initial volume of the vital cell corona, $V_{v}^{i}$, which contains the normoxic and the hypoxic cells, is given by

(A16) $$V_{v}^{i}=V_{T}^{i}-V_{n}^{i},$$

and therefore

(A17) $$V_{c}^{i}=V_{v}^{i}-V_{h}^{i}.$$

The treatment here discussed is based on the localization of five vertices as in Figure 1, and the vertex volume can be written as the sum of the overlap, $v_{1}$, with the necrotic core and of the overlapping volumes, $v_{2}$ and $v_{3}$, with the hypoxic and normoxic spherical coronas. The subvolumes $v_{1},v_{2},v_{3}$ can be easily evaluated by the geometrical formula for the two-sphere overlap.

The initial values are $V_{T}^{i}=171.3$ cm^3^, $V_{n}^{i}=86.8$ cm^3^, $V_{h}^{i}=13$ cm^3^ and 0.4 cm^3^ for each vertex. The radius of the necrotic core turns out to be 2.75 cm, the thicknesses of the hypoxic corona and of the normoxic corona are, respectively, ≃0.14 and ≃0.56.

The vertices receive a first dose of 15 Gy, followed by the standard treatment of ten daily doses of 3 Gy, uniformly distributed in the whole tumor volume. Moreover, the effects of the initial large dose in the vertices on the other vital cell areas, due to radiation distribution, must be taken into account. The average dose delivered to the normoxic cell turns out to be $d_{nor}=4.3$ Gy and $d_{hy}=5.1$ Gy for hypoxic cells.

According to the previous discussion, applying the LQ model and the OER reduction, the various volumes after the first dose, *D*, of 15 Gy and before the standard treatment (ten daily doses of 3 Gy) are given by:

(a) Normoxic volume

(A18) $$V_{nor}^{in}=\left(V_{c}^{i}-5.0v_{3}\right)e^{-(\alpha d_{nor}+\beta d_{nor}^{2})}+5.0v_{3}e^{-(\alpha \times D+\beta \times D^{2})};$$

(b) Hypoxic volume

(A19) $$V_{hy}^{in}=\left(V_{h}^{1}-5.0v_{2}\right)e^{-(\alpha_{h}\times d_{hy}+\beta_{h}\times d_{hy}^{2})}+5.0v_{2}e^{-(\alpha_{h}\times D+\beta_{h}\times D^{2})}.$$

By initial values and the geometrical evaluation of $v_{1},v_{2},v_{3}$, one gets $V_{nor}^{in}≃20.7$ cm^3^ and $V_{hy}^{in}≃5.05$ cm^3^, with ≃70% and ≃60% of reduction of the corresponding initial (no treatment) volumes.

The initial dose and the standard protocol activate an immune response. Let us initially neglect this effect (i.e., M(t)=0 in Equation (A12)) and the very small contribution due to the regrowth (i.e., k=0 in Equation (A5)). The final volumes after *n* daily doses, d=3 Gy, obtained by the LQM are given by

(A20) $$V_{c}^{f}=V_{nor}^{in}e^{-[\left(\alpha d+\beta d^{2}\right)]n},$$

(A21) $$V_{h}^{f}=V_{hy}^{in}e^{-[\left(\alpha_{h}d+\beta_{h}d^{2}\right)]n}.$$

The survival fractions, obtained by the ratio of the previous volumes with the corresponding initial ones, for n=10, α=0.2, β=α/10, are reported in Table A1 for the normoxic area. The final volumes of the active areas are very small compared to the final tumor volume (113.1 cm^3^) and essentially undetectable by metabolic activity (PET).

**Table A1:** Survival fraction for normoxic cell evaluated by LQM with vertices and 3 Gy for 10 days; 0 shows the survival fraction after the initial large dose and before the standard treatment.

Day	Reduction Factor
0	0.29
1	0.13
2	$6.1\times 10^{-2}$
3	$2.8\times 10^{-2}$
4	$1.3\times 10^{-2}$
5	$5.9\times 10^{-3}$
6	$2.7\times 10^{-3}$
7	$1.23\times 10^{-3}$
8	$5.7\times 10^{-4}$
9	$2.6\times 10^{-4}$
10	$1.19\times 10^{-4}$

However, by assuming a constant density of $10^{12}$ tumor cells in a liter [12], the number of active cells is still quite large. Therefore, a recurrence is rather possible. On the other hand, before discussing the critical condition for stable disease, complete recovery or tumor regrowth, one has to include the effect of the immune response activated by radiotherapy, which at the end of the ten treatments is given by by the term A(n=10).

Its evaluation requires an explicit model of the function M(t), i.e., a microscopic dynamical model. Independently of the specific models, let us assume that the induced response remains almost constant after the end of radiotherapy since the immune system has been already activated. In other terms, after the final dose, the tumor progression follows the evolution law (in terms of the cell number *N* and *t* in days)

(A22) $$N\left(t\right)=N\left(n\right)e^{ln\frac{N_{\infty }}{N(n)}\left[1-e^{-k(t-n)}\right]-A\left(n=10\right)}\;\;t\geq n$$

According to the previous assumption, the immune-activated response decreases the number of proliferating cells at the end of radiotherapy, i.e., N(n), and contributes to the progression pattern for t≥n. Therefore if

(A23) $$ln\frac{N_{\infty }}{N(n)}-A\left(n=10\right)<0,$$

one gets a complete recovery.
